# Nasopharyngeal carcinoma ecology theory: cancer as multidimensional spatiotemporal “unity of ecology and evolution” pathological ecosystem

**DOI:** 10.7150/thno.82690

**Published:** 2023-03-05

**Authors:** Weiren Luo

**Affiliations:** Cancer Research Institute, Department of Pathology, The Second Affiliated Hospital of Southern University of Science and Technology, Shenzhen Third People's Hospital, National Clinical Research Center for Infectious Diseases, Shenzhen, China.

**Keywords:** Nasopharyngeal carcinoma ecology, Unity of ecology and evolution, Pathological ecosystem, Tumor microenvironment, Tumor-host interface, Tumor budding, Ecological therapy, Ecological pathology, Ecological radiology, Synthetic cancer ecology, Multidimensional tumoriecology, Cancer ecology tree

## Abstract

Nasopharyngeal carcinoma (NPC) is a particular entity of head neck cancer that is generally regarded as a genetic disease with diverse intertumor and intratumor heterogeneity. This perspective review mainly outlines the up-to-date knowledge of cancer ecology and NPC progression, and presents a number of conceptual stepping-stones. At the beginning, I explicitly advocate that the nature of NPC (cancer) is not a genetic disease but an ecological disease: a multidimensional spatiotemporal “unity of ecology and evolution” pathological ecosystem. The hallmarks of cancer is proposed to act as ecological factors of population fitness. Subsequently, NPC cells are described as invasive species and its metastasis as a multidirectional ecological dispersal. The foundational ecological principles include intraspecific relationship (e.g. communication) and interspecific relationship (e.g. competition, predation, parasitism and mutualism) are interpreted to understand NPC progression. “Mulberry-fish-ponds” model can well illustrate the dynamic reciprocity of cancer ecosystem. Tumor-host interface is the ecological transition zone of cancer, and tumor buddings should be recognized as ecological islands separated from the mainland. It should be noted that tumor-host interface has a significantly molecular and functional edge effect because of its curvature and irregularity. Selection driving factors and ecological therapy including hyperthermia for NPC patients, and future perspectives in such field as “ecological pathology”, “multidimensional tumoriecology” are also discussed. I advance that “nothing in cancer evolution or ecology makes sense except in the light of the other”. The cancer ecology tree is constructed to comprehensively point out the future research direction. Taken together, the establishment of NPC ecology theory and cancer ecology tree might provide a novel conceptual framework and paradigm for our understanding of cancer complex causal process and potential preventive and therapeutic applications for patients.

## Introduction

Nasopharyngeal carcinoma (NPC) is a disease with remarkably distinctive ethnic and geographic distributions, which is highly prevalent in Southern China and Southeast Asia. The pathogenesis of NPC is closely associated with Epstein-Barr virus (EBV) infection, other pathogen infections such as HPV and bacteria and environmental factors including chemical carcinogens and social practice such as alcohol consumption and smoking also contribute to the increased risk of NPC 1-4. Although a large number of funds, labor and material resources have been spent in the last decades, a brutal truth is that most of patients are still diagnosed at advanced stages, and tend to have local recurrence and distant metastasis after conventional therapy, even emerging immunotherapy such as PD-1 inhibition benefits only a subset of clinical cases 5. Perhaps, it is time for us to rethink this disease viewed as the characteristic cancer in China. But where to move on? This is what this paper is about.

NPC is generally characterized as a genetic disease with diverse extent of intertumor and intratumor heterogeneity 6. During the past several decades, a great deal of attention and achievements have been reached according to the possible mechanisms about the genetic and molecular carcinogenesis and metastasis of this disease, as well as the mutual regulation of the molecules such as DNA and RNA (e.g. miRNA and lncRNA). However, we should be aware that the role of DNA is not for programming cells. As early as in 1962, the radiologist Smithers DW has stated that "cancer is no more of a disease of cells than a traffic jam is a disease of cars" 7. Too much concentration on internal combustion engines would not help us to understand the traffic problems. NPC is a complex social community. It consists of dysfunctional cell populations and a great heterogeneous context that is characterized by highly variable immune cells infiltration such as lymphocytes and plasma cells (some cases with strong desmoplastic stroma) 8,9. Concentrating on what goes on within a tumor is certainly insufficient for elucidating the underlying mechanisms that support malignant spread and metastasis. In case taking a part for the whole, we should acknowledge that the impacts of tumor microenvironment (TME) on NPC have gained much more attention over the past ten years 10. However, it is not difficult to find such a fact that there was a lack of the matrix context and spatial architecture in most *in vitro* experiments. Additionally, I think that the complex relationship between NPC cells and TME also needs to be generalized essentially.

Su Shi, a famous poet in the Song Dynasty of China, once wrote “it's a range viewed in face and peaks viewed from the side; assuming different shapes viewed from far and wide”. Therefore, it is necessary to turn this disease from reductionism to holism, and ultimately reach out unity of molecular reductionism with quantitative holism. A multidimensional perspective is needed to review the historic research of NPC and its microenvironment, and to clearly elucidate the nature of this disease and move cancer research into a new direction. In this study, I propose that NPC is significantly more complicated than “a genetic disease”, and it could best be conceptualized as a multidimensional spatiotemporal “ecological and evolutionary unity” disease: an evolutionary adaptive pathological ecosystem that consisting of four interdependent parts: primary ecosystem, circulating ecosystem, metastatic ecosystem and multidirectional ecosystem, which was predescribed on 17 October 2022 (https://www.preprints.org/manuscript/202210.0226/v1). The ecological theory of NPC is hoped to provide a novel and fundamental framework about comprehensively understanding of the complicated progression of this disease, and develop more effective treatment strategies for cancer patients.

## NPC as pathological ecosystem of “unity of ecology and evolution”

The first use of the word “ecosystem” began with British ecologist Tansley AG in 1935 11. In the 1950s, Odum EP creatively proposed the change rules of the structure and function characteristics during the ecosystem development as “any unit that includes all of the organisms (the biotic community) in a given area interacting with the physical environment so that a flow of energy leads to clearly defined biotic structures and cycling of materials between living and nonliving components is an ecological system” 12. A nature ecosystem consists of two main parts including populations (animals, plants, virus, bacteria, etc.) and the physical environment they inhabit (oxygen, water, soil, food, etc.). Similarly, cancer tissues including NPC create a complex spatially structured ecosystem made up of various cell types and essential stromal resources. Microscopically, NPC cells encompass squamous cell carcinoma, nonkeratinizing carcinoma (differentiated or undifferentiated) and basaloid squamous cell carcinoma. Among them, differentiated carcinoma (DNKC) and undifferentiated carcinoma (UDC) are the main cell subtypes. It should be noted that either the differentiated or undifferentiated subtype generally has a certain number of spindle cells (sarcoma-like) 8,13, which tend to cause erosion of surrounding tissue 9 and predominantly present in the invasive front or infiltrating the stroma 14,15. On the other side, the NPC microenvironment is highly heterogeneous with tumor stroma reaction 9, biotic components comprise of cancer-associated fibroblasts (CAFs), immune cells and endothelial cells, and abiotic components include extracellular matrix (ECM), cytokines, nutrients 8,12,16. Together, we can postulate that this variety of cancer cell subtypes, cooperating and competing with each other and interacting within their habitats spatiotemporally in the whole ecosystem of a high degree of genetic and phenotypic diversity, that jointly contribute to both malignant progression and clinical practise in NPC patients.

It is well-known that in 1973, Dobzhansky T made a remark as “nothing in biology makes sense except in the light of evolution” 17. It is a truism not just for biology but also for many disciplines of medicine like oncology 18. During the pathological diagnosis of nasopharyngeal biopsy specimens, the changes from low or high dysplasia to carcinoma-in situ (the prevalence varies from 2% to 3.6% of NPC) can be observed in the place where is jointed with normal nasopharyngeal epithelial cells. Histologically, these atypical cells consist of cells with variable loss of polarity, or enlarged oval-shaped pleomorphic nuclei with obvious nucleoli and indistinct cytoplasmic boundaries confined to the surface or crypt epithelium 12,19. There is a meaningful implication of carcinogenesis from the normal nasopharyngeal epithelium. It is generally accepted that nasopharyngeal epithelial transformation and pathogenesis is closely linked with, at first chronic exposure of the nasopharyngeal mucosa to environmental carcinogens such as nitrite amines, alcohol consumption and smoking, and then accelerated by the infection of EBV infection (other pathogens such as HPV and bacteria might also be involved) 1-5. As the early stage of initiating tumorigenic transformation, for example, reports have showed direct infection and transformation of human nasopharyngeal epithelial cells destined to evolve into the carcinoma by EBV through EBVR/CR2 20. EphA2 is also a critical player for EBV epithelial cell entry 21. In addition, EBV-infection encoded oncoproteins such as LMP1, LMP2 and EBNA1 or their interactions with the TME includes immune cells and rodent fibroblasts have been proved to directly impact morphological and phenotypic alterations in epithelial cells, and the malignant progression or therapy response of NPC 22-25. One of the best models of the roles of EBV infection and genomic changes during NPC development was constructed 5. In fact, as early as the 1980s, Yao KT has advocated the “three strikes” hypothesis to interpret the pathogenesis of NPC 2. In brief, some familial cases often carry germline mutation, also known as reproductive mutation (one strike), and the combination of EB virus (two strikes) and environmental factors (three strikes) eventually leads to the carcinogenesis of this disease. These abnormal cells require the sequential acquisition of mutations from the prospective of Darwinian evolution “survival of the fittest” 26, with altered metabolic, mechanical, and cell communication contexts that act as ecological stress factors. Under these circumstances, cells need to compete with each other for survival, and thus the fittest cells can survive and form genetically distinct cell populations, the tumor. To summarize, I conclude that progressively transformed of normal cells to carcinogenesis could be viewed as an ecol-evolutionary process.

In the 1970s, Peter Nowell firstly described a landmark perspective on dynamic tumor evolution and progression 27. This theory implies that increasingly aggressive subclones in cancer cell populations are selected to evolve according to the Darwinian principles, which results in genetically heterogeneous tumors (intertumor and intratumor heterogeneity) and is potentially responsible for cancer formation, drug resistance and metastasis 28. Over the past decades, based on the advancing technologies such as microarray technology, genome-wide analysis and whole-exome sequencing, tumor heterogeneity, intraclonal genetic diversity and high genomic instability in NPC have been widely excavated and disclosed 29-31. These empirical findings should be the basis of portrait of genetic architectures and inferred clonal evolutionary trees. In recent few years, the application of single-cell transcriptomics has contributed to a more comprehensively and attentive understanding of the stromal landscape and immune dynamics and their interplay with cancer cells in the whole NPC 32-35. Temporal changes of the microenvironment's heterogeneity including enrichment of M2-polarized macrophages and LAMP3+ dendritic cells between the primary and recurrent NPC (rNPC) has been revealed 36. On the basis of the Darwinian principles 37,38, we should realize that, the heterogeneity of NPC could be the result of competition between various clones of cancer cells that act as competing species for resources in the tumor microenvironment. In turn, the subpulolations of NPC cells could acquire, through mutational and epigenetic changes, a variety of phenotypic traits that compound to allow territorial expansion by proliferative self-renewal, diaspora and therapeutic resistance.

**Figure 1:**
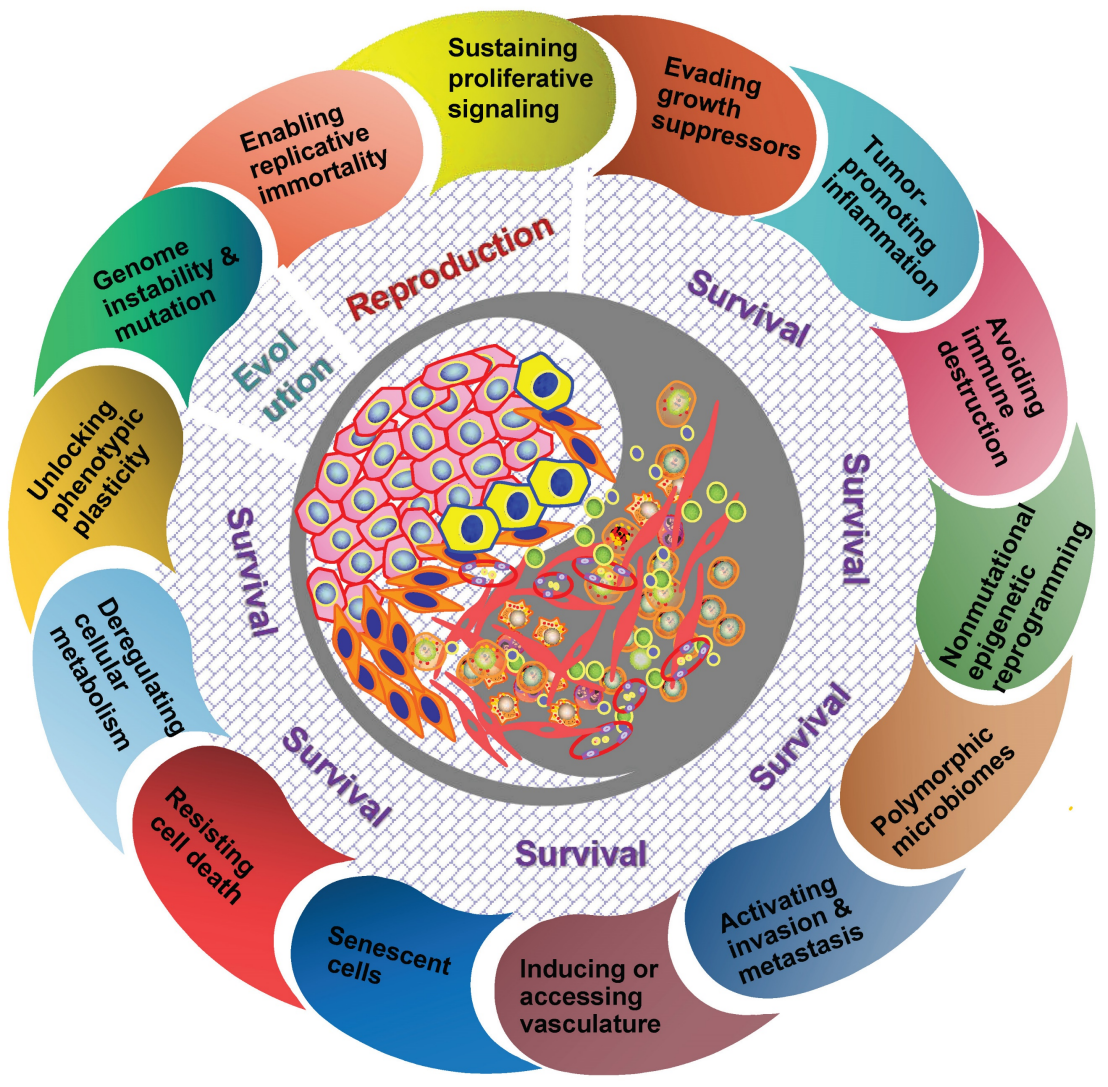
The hallmarks of cancer act as ecological factors of population fitness. Genome instability and mutation lead to the diversity and evolutionary adaptation of population species, other cancer hallmarks affect ecological adaptation of cancer cells through the dynamic change of their survival ability and reproductive capacity in particular.

It should be understood that diverse clones of cancer cells need not to be genetically encoded. Tumor microenvironment has been proved to be a major determinant of tumor heterogeneity 39. The ecological interaction between cancer cells and their habitats is dynamic reciprocal. A tumor can be regarded as being an integrated ecosystem in which the co-evolution of neoplastic cells together with tumor microenvironment such as extracellular matrix, tumor vasculature and immune cells confers a highly fitness to exceptionally variable phenotypes of cancer cells 40,41. The selective advantage may be achieved by various fitness phenotypes (or ecological factors) so-called “hallmarks of cancer” , which includes the acquired capabilities for sustaining proliferative signaling, evading growth suppressors, resisting cell death, enabling replicative immortality, inducing/accessing vasculature, activating invasion and metastasis, reprogramming cellular metabolism, avoiding immune destruction, unlocking phenotypic plasticity, nonmutational epigenetic reprogramming, polymorphic microbiomes, and senescent cells 42. So the point I'm emphasizing here is that the great majority of these hallmarks ultimately affect cancer cell fitness through the ability of survival and/or reproduction (**Figure 1**). Most of hallmark phenotypic features governed by the crosstalk between cancer cells and tumor microenvironment in NPC have also been explored in more recent years 8,16,32-36,43,44. Here I'd like to emphasize that, “nothing in evolution makes sense except in the light of tumor microenvironment”.

Tumor therapeutic resistance, relapse and distant metastasis is essentially an evolutionary process 45,46. Many cancers, if not all, a small subpopulation of cells in tumors termed as cancer stem cells (CSCs) should be the representative for elucidating this evolutionary process because of the capacity of maintaining tumor heterogeneity, unlimited proliferation and evading therapeutic predation 47,48. On the other side, CSCs can proactively remodel the surrounding tissue microhabitats to maintain a perivascular niche and phenotypic variation in fitness (that is, ecological engineering and niche construction) 49. During the past decades, diverse identifying approaches and experiments have been applied for the study of CSCs in NPC 50. The presence of CSCs in NPC was initially identified by label-retaining cell (LRC) approach by Yao KT et al. in 2007 51. Subsequently, Zeng YX et al. reported that side population (SP) cells of NPC had stem cell-like properties with an enhanced ability to form tumors* in vivo* 52. Our previous study also showed that aldehyde dehydrogenase 1 (ALDH1) could be a novel CSCs marker in NPC, which was endowed with extensive proliferation, capable of self-renewal and generating tumors 53. Moreover, CSCs themselves are found to be overrepresented at the invasive front (tumor-host interface) of NPC tissues. For example, EBV-encoded LMP2A, FoxM1 could enhance the ability of tumorigenicity of NPC cell lines in mice xenograft, and the expressions of these proteins were mainly localized at the tumor invasive front 54,55. As such, CSCs at the invasive front acting as an driver might contribute greatly to metastatic features and worse prognosis of NPC patients.

The same species may generate different morphological structures and physiological characteristics under different conditions in which they live for a long time, and this type of biological adaptation is called “ecotype” 56. The morphological and/or physiological attributes are generally caused by the selection of habitat environment 57-60. The cancer cells in NPC specimens show light microscopic evidence of squamous differentiation, and their microenvironment comprises the very complex stromal components. As mentioned above, either the differentiated or undifferentiated subtype generally contains a proportion of neoplastic spindle cells, and these cells with fibroblast-like phenotype are observed predominently in the invasive tumor front. 14,61. Given such communities of species and their dynamic interactions with each other and their environments day and night, it is reasonable to assume that spindle cells are reflection of morphological adaptation to the external environment from squamous subtypes. In view of these cells possessing the epithelial-mesenchymal transition (EMT) and CSCs features, it might be considered as the morphological indicators of “mobile/migratory CSCs” and “invasive/metastatic NPC” 61. Additionally, vasculogenic mimicry, a new tumor microvascular paradigm of non-endothelial cells, is formed to adopt multiple cellular phenotypes including tube-like structure or endothelial-like properties, and contributes significantly to the progression and metastasis of NPC 62-65. On the other side, different populations also could display the same or similar adaptive characteristics through variation and selection under the long-term living in the same habitat 56. It is noteworthy that in some cases, NPC cells are quite similar to the morphology of benign cellular changes such as clusters of germinal centre cells and reactive lymphoid hyperplasia 11,12. In clinical practice, the computational application and deep learning model have been recently used by us and other groups for the successful identification 66-68, and largely assist pathologists on these difficult diagnoses 68.

What any ecologist sees is the result of evolution. Any script of evolution is staged on the stage of ecology. Now Dobzhansky's famous dictum has been taken on a whole new meaning “nothing in evolution or ecology makes sense except in the light of the other” by Pelletier F in 2009 69. The same is true for human neoplasm. I advance here that, “nothing in cancer evolution or ecology makes sense except in the light of the other”, both of cancer evolution and ecology are interdependent to each other and mutually promoting. In a word, the essence of NPC as well as other kinds of human neoplasms is “unity of ecology and evolution”.

## NPC as invasive species and its metastasis as an ecological dispersal

Population dispersal generally means that some individuals leave the original habitat and spread out 70-74; for example, terrestrial birds living in an archipelago seek a suitable habitat among different islands. The diaspora of NPC shares several common features with the ecological concept of “diaspora”. NPC metastasis can be thought as a form of ecological dispersal owing to invasive species destroying the local ecosystem and setting up a base in distant organs through their proliferation and spread after entering the circulation to undergo jump dispersal. One of the critical characteristics of a diaspora is that dispersed populations retain a sense of their uniqueness that maintains collective memory of their original habitats 75,76. This is mainly reflected by the establishment of a new habitat, and maintaining a identity as the same as their original homelands. NPC patients tend to have cervical lymph node metastases at early stage, and metastasize to bones, lungs, livers and other organs after therapy 4-6. In most of samples, the microscopically histopathological type of metastatic sites is consistent with primary cancer cells, whereby pathologists can easily identify metastatic NPC that is originated from the original tumors. On the other side, most of molecular characteristics of metastatic sites and those of primary tumors show no significant difference in many pathological cases.

Another critical aspect to understand the feature of biological diaspora is that native environments and homeostasis always are disrupted by invasive species 77. Whether in the primary sites or metastatic areas, the external environment is generally hostile to the invaders. These malignant cells confront with many physical barriers before they can invade, survive and favor the new environment for further colonization and dissemination. Consistent with the “seed and soil” theory of metastasis proposed by Paget in 1889 78, the best predictors of the success of ecological dispersal are those variables that whether cancer cells have enough ecological adaptive and reproductive ability 79-81. In the primary sites, for example, NPC cells can secrete matrix metalloproteinases that physically alter their environments 82-84, contribute to angiogenesis via vascular endothelial growth factor (VEGF) production 85-87, manipulate the content and functions of extracellular vesicles (EVs) 88-92 or change tight vascular capillary endothelial walls by inducing endothelial-mesenchymal transition (EnMT) 93 of the organ ecosystem. This is well reflected in the detection of magnetic resonance imaging (MRI) with extensive local infiltration of adjacent soft tissues, erosion of skull base, etc. If observing through the microscope, we can find that the normal tissue architecture (e.g. lymph nodes, vascular architecture) is destroyed by clusters of cancer cells.

Jump dispersal refers to the fast movement of population species over a long distance, and then the establishment of individual organisms at the destination, for instance, the spreading of Argentine ants throughout the United States 94-96. Tumor cell metastasis through the circulation is a great example of jump dispersal 78,79. In the ecological system of Nature, multiple organisms encounter different kinds of abiotic or biotic barriers on their journey, such as the dispersal of shallow-water marine species is hampered by stretches of deep ocean constitute barriers in the tropical Pacific. Likewise, CSCs also encounter such dispersal barriers during their translocation to distant organs/tissues. Such according process can be grouped roughly under the following points: 1) The diameter of CTCs is three to four times that of the capillary pores in distant organs. So they would be trapped there before extravasating from blood and lymphatic vessels 97,98; 2) In the circulation system, these CTCs confront with a rather harsh habitat completely different from the original sites, where they are susceptible to anoikis and mechanical stress, and vulnerablely attacked by immune cells such as NK cells in the circulating 99,100. The latest study demonstrates that platelet-derived RGS18 protects CTCs from NK-mediated immune surveillance by hijacking immune checkpoint HLA-E:CD94-NKG2A 101; 3) When cancer cells reach the distant sites, they have to adapt to the new microenvironment such as ECM components and the immune system seem to don't welcome their arrivals 102. These disseminated tumor cells (DTCs) need to construct the local conditions as “ecological engineers” to set up their habitat to assist the survival 79-81,103, that is, creating pre-metastatic niche (niche construction) such as immunosuppression, tumor-promoting inflammation, inducing angiogenesis and ECM reprogramming, and aerobic glycolysis producing an acidic local environment 104-106. For instance, they attract angiogenesis vasculature to ensure a supply of oxygen and nutrients, and switch energy metabolism to glycolysis for hypoxia adaptation 107,108. Therefore, only the most adaptable phenotypes of CTCs could be successful to root in distant organs. The dynamic ecology of DTCs and the pre-metastatic microenvironment can be “pre-metastatic ecosystem”.

Let's briefly have a look at some advances of CTCs research in NPC. The first report about the capture of CTCs in peripheral venous blood was identified by CK-19 positive cells in NPC 109. Our previous study indicated that dissociated NPC cells in the circulation exhibiting EMT/CSCs features were endowed with their metastatic potentials 61. CTCs can be used for tumor stage determination and prognosis prediction of NPC at all stages, and especially epithelial/mesenchymal (E/M) hybrid CTCs predict adverse outcomes 110,111. To be more exhilarating, a study showed that CTCs can be a novel more sensitive biomarker for minimal residual disease (MRD) of NPC patients 112. As yet, we should realize that the area of “circulating ecology” and “metastatic ecology” in NPC remains largely undisclosed, for example, what is the different immune-tumor ecological interactions in primary, circulation and metastatic lesions, it is worth of further investigation.

Dormancy is an organism's ability to enter a dormant state of low metabolic activity when facing with adverse environmental conditions 113-115, as such increases fitness by delaying the time in the habitat. Likewise, a phenomenon called "tumor dormancy" is very common in clinical practice, that is, disseminated cancer cells can seed in the host tissues for years before the local recurrences or metastases can be detected 116,117. Tumor dormancy is considered as a conserved evolutionary process related to adaptation and resistance to selective stress that is imposed by dissemination and the hard microenvironment within the target organs 118-121. Up to now, molecular mechanisms of cancer dormancy have been widely investigated. For example, Weeraratna AT et al. reported that aged lung microenvironment can facilitate a permissive niche and promote the efficient reactivation of dormant melanoma cells in the lung through WNT5A, AXL and MER activators 122. For the dormancy of NPC, a study from Chinese Taiwan reported the high susceptibility of primary NPC cells following transferred IFN-γgene to the induction of tumor dormancy 123. Three-dimensional culture studies suggest that the LTBP-2 contribute to tumor cell dormancy in a growth factor favorable microenvironment 124. However, we have to admit that the microenvironment signals regulating the dormancy of NPC still remain poorly understood by now, which might hamper our fully understanding of the complexity of the disease.

It is generally accept that the metastasis of NPC is a complex, multifactorial and multi-step process. However, for the spatial knowledge about the recurrence and metastasis of NPC, we may often stay in the simple “one-way migration” mind, that is one-way trip “A to B”. Can this progression be thought at least as double trip “A to B then to A”? A great challenge in clinical practice is that distant/recurrent metastasis contributes to the major reason for therapeutic failure and most of these cancers are still incurable 75,97,125, that's really the main question before us. This harsh fact might indicate that conventionally describing metastasis as a simple one-way migration of cells from the primary sites to target organs is too far behind to elucidating the complex progression of cancer. One remarkable feature of population dispersal is that emigrants dispersing to habitat patches connect with the host each other through exchange of individuals and information. Indeed, cancer cells can seed distant sites as well as the primary tumor itself, and this phenomenon is so-called "self-seeding” 126,127. The “self-seeding” demonstrates that metastasis is a bidirectional process whereby cancer cells can seed the primary tumor itself by CTCs. This metastatic hypothesis has been validated in experimental models of breast cancer, colon cancer and melanoma 128. Exploiting clinical investigation within a self-seeding model may offer new possibilities for eradicating metastatic cancer. Unfortunately, a view of "self-seeding" process of NPC has not yet been proposed before. Currently, management of local recurrent NPC remains one of the most difficult challenges 129, and the related factors remain largely unknown. Emerging studies have showed that the local residual cancer cells (i.e. CSCs) might be responsible for this problem. Do self-seeding of CTCs or metastatic tumor cells at distant sites also work, or can their releasing soluble factors such as exosomes, cytokines, chemokines and growth factors self-feedback? On the other side, for patients with metastasis, can these metastatic population cells cause a second metastasis in other organs? From the perspective of the human body as the entire biosphere, this entirely makes sense. Two case reports as below can give us more or less such hints. One case is that this patient carried liver metastasis three months after clinical complete response, and after Transcatheter Hepatic Artery Chemoembolization (TACE) treatment, the liver metastases maintained stable for six months, but lung metastases were found to be established 130. Another case with occipital lymph node metastasis developed multiple distant metastases in the sternum, left ilium, bilateral internal mammary lymph nodes and spleen, while the nasopharynx and related regions remained well controlled without sign of recurrence 131. Considering the therapeutic complexity of NPC, the multidirectional process might reshape our thinking of cancer metastasis, and offer alternative opportunities for prevention and therapy of recurrent/metastatic NPC. Meanwhile, it should be noticed that we have a long way to go, more convincing data needs to be validated through the integration of mathematical, experimental and animal models.

**Figure 2:**
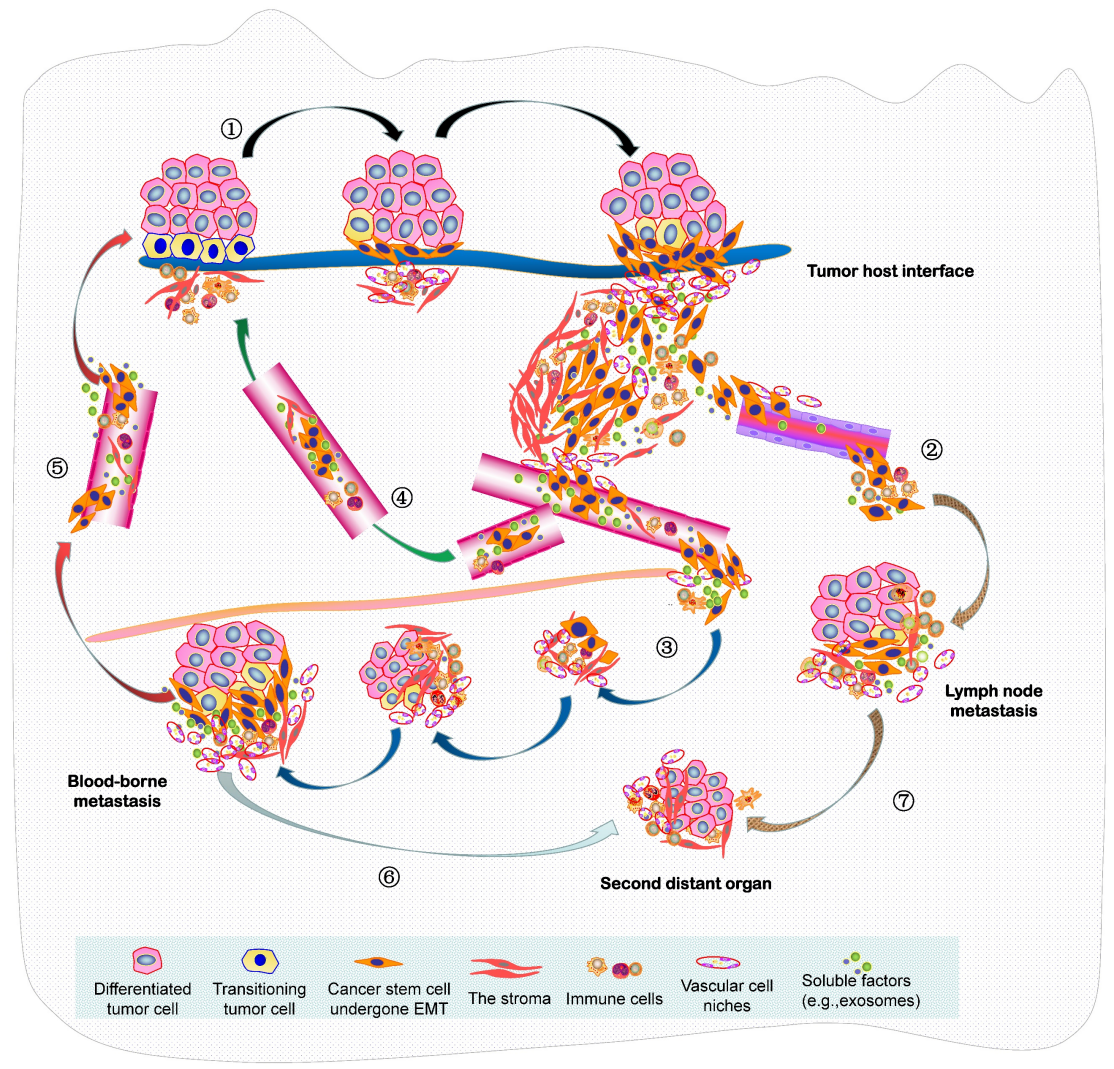
A novel ecological dispersal model of tumor multidirectional progression in NPC is proposed. During this process, ① NPC cells with CSCs characteristics undergo spindle-like phenotypes through EMT (mainly adapt to the selective pressure from the remodeling microenvironment) to dissociate from tumor-host interface (e.g. budding cells) and interplay with the various stroma components (*local primary ecosystem*); intravasate into the circulation (through either ② lymphangion or ③ blood vessels), survive the stresses of the circulating process, and extravasate to a metastatic site (lymph node or distant organ) (*circulating ecosystem*); enter slow-cycling states for dormancy, escape immune predation, engineer organ-specific niches to colonize micro/macro-metastases and later spread (*distant metastatic ecosystem*); Self-seeding of ④ CTCs or ⑤ metastatic tumor cells at distant sites, or their releasing soluble factors such as exosomes, cytokines and chemokines (self-feeding), or host cells include CAFs and immune cells (self-accomplice) return to primary tumor (*multidirectional ecosystem*) ; Additionally, metastatic cancer cells at ⑥ distant organ or ⑦ lymph node can produce the new populations in the second distant site.

In summary, the seed and soil hypothesis indicates that the metastasis of NPC is analogous to the colonization of a new habitat, and this invasive and evolving progression should be better recognized as a form of ecological dispersal. Resulting from the increasingly limited space, high density, hypoxia and low nutrients within a tumor mass, this undoubtedly leads to fierce competition of cancer cells, there will then be selection for invasive species to facilitate the diaspora that can be seen as cell populations maximizing their average fitness. Such malignant progression is indeed “ecological invasion” (invasion ecology). In 2013, we constructed an integrated molecule-morphology model to elucidate the metastatic cascade of NPC 61. At the basis of it, a novel model about ecological dispersal of NPC progression is depicted here (**Figure 2**). The whole ecosystem of NPC can be classified into four interdependent parts: local primary ecosystem, circulating ecosystem, distant metastatic ecosystem and multidirectional ecosystem. I suggest that these classifications of ecosystems are not chaotic cancer cell masses, they could be dynamic cross-level organization in time-space manner. In order to promote survival rate and produce more offspring, various groups of tumor cells in the whole ecosystem interact (short- or medium- or long-range interactions) and communicate “intellectually” with each other and a complex microenvironment, they respond quickly to the stimulation of external factors and make the optimized decision through ecol-evolutionary pathways within cellular networks or intercellular networks. Their ecol-evolutionary adaptation courses are an exceptionally interesting area of future study.

## Exploiting ecological principles to understand NPC progression

The population in ecology is defined as collection of individuals of the same species with unique characteristics coexisting at the specialized time and place 132. Population is an important unit of intertwined ecological and evolutionary process. Similarly, cancer cell population is considered as collection of subpopulations of cancer cells with different survival and reproductive abilities living together at regions of spatial heterogeneity within a tumor 37,133. The variation among individuals within a population is widespread. These differences are determined by a combination of specific genotypes and environmental influences. As described above, NPC is a complex ecological disease because these cancer cells are spatially and temporally heterogeneous, they continually interact and even evolve with each other and the microenvironment in order to increase the fitness of the cancer cells. In general, the malignant progression of NPC contains two parts of intraspecific relationship and interspecific relationship. The basic ecological principles to understand and manage this disease are exploited as below.

Intraspecific relationship refers to the relationship between individuals within a population, which can be classified by the fitness effects of the individuals (neoplastic cells) on each other includes coexistence, competition, spatial behavior and communication behavior 45,134,135. As to specie coexistence, for example, the environment offers different types of foods or resources, and diet choice provides the most basic mechanism of coexistence. The ideal example of resource partitioning via diet choice hails from the Galapagos Islands. Different species of ground foraging Darwin's finches partition seeds according to the shape of the bird's beak and the size of the seed 132. Microscopically, the coexistence of two cell subtypes including DNKC and UDC is generally present in NPC tissues. In some cases, a certain content of spindle cells that might be the morphological characteristics of CSCs is also found. The cytokines secreted by cells within a tumor and abiotic environmental factors such as amino acids and trace nutrients provided through the blood consist of the diverse and heterogeneous pool of foods that could promote coexistence of different cell subtypes of this disease. Experimental observation under the microscope can find that the coexistence and proportion of these cancer cells are closely related to the surrounding environment such as the formation of tumor angiogenesis. The vascular niche is closely interacted with stem cell-like NPC at the invasive front 136, which has been proved to be crucial to tumor migration and metastasis by secretion of various angiocrine factors 104,137,138. All coins have two sides. As tumor cells divide, they can outstrip their blood supply, and create a hypoxia conditions, nutrient poor and acidic stagnant swamp rather than a healthy pond 139-141. The lysate of necrotic cells and the release of pro-inflammatory cytokines secreted by cancer cells combine to produce equal amounts of swamp gas, this model is so called toxic “cancer swamp” 141. The cancer swamp is exactly the environment in which cancer cells create a harsh habitat that will exert selective pressure and generate the adaptive clones with the abilities to migrate and metastasize. Interestingly, a novel imaging strategy was recently exploited for quantitative 3D evaluation of hypoxia heterogeneity in an orthotopic model of NPC 142.

Due to the diversity of natural environments, and the competition among individuals within the species, different populations can exhibit different spatial behaviors 143. In most situation, the populations are often distributed in groups. Commonly, collected distribution has greater resistance to adverse environment, meanwhile it also increases the competition among the individuals. Reproduction success rather than survival is the main criterion to measure biological adaptation of cancer populations. Competition for the limited space and resources deceases densities and population sizes of cancer cells. When it becomes harsh microenvironment like hypoxia and acidic conditions, better adapted populations will win the competition and grow in the invasive phenotypes 144,145. And that's clearly true here. Spatial behaviors of NPC microscopically are generally present with the format of “collective migration” including but not limited to the center of tumor mass displaying solid sheets, irregular islands or dyscohesive sheets. In contrast, collective movement of neoplastic cells at the tumor-host interface or in the stiffening stroma of NPC is often less ordered and has more mesenchyme-like phenotype.

Population communication is that an individual releases one or several stimulus signals, sends information, then another individual receives information and initiates specific behavior. Among them, chemical communication is a very common behavior for calling together with each other and acting together in groups. Intercellular communication through extracellular vesicles in cancer and evolutionary biology has been widely demonstrated 146-149. A recent report has found that EVs transferred from highly to poorly metastatic NPC cells could mediate cell-cell communication and enhance the metastatic potential of poorly metastatic cancer cells 150. In addition, an intriguing phenomenon is that cancer cells can move through an epithelial bridge, and seem to enable a new form of cell communication and migration (http://ki-galleries.mit.edu/2012/zani). Such communicating bridge was also observed among breast cancer clusters when I was working in Bissell MJ's lab at LBNL as visiting scholar. By on-top 3D cell culture, two separated cancer islands were found to be connected completely by the bridge (data not shown). It might be the novel communicating way for the direct transform and exchange of energy and message in cellular cancer ecosystem.

Interspecific relationship means that the interaction between the different species, which essentially includes interspecific competition, predation, parasitism, mutualism, etc. 10,132,151. These different ecological interactions can be found in various kinds of neoplasms including NPC, and that are key regulators of tumor ecosystem. Owing to technological advances, it has been clear that intercellular communication between cancer cells and stromal cells is established by extracellular vesicles including exosomes and ligand-receptor interactions, and other mechanisms involving cytokines and chemokines 152-154.

Predation is defined as one species attacks or kills another species and feeds on it. The former calls a predator, and the latter is a prey 135,151. Population predation might be best applicable to the interaction of neoplastic cells with the immune system. We must be aware that the research on immune cells of NPC has a long history. Since the 1970s, lots of efforts have been made to evaluate the expression levels of immune cells and their prognostic values 155-160 by immunohistological analysis, flow cytometry or other experimental methods in NPC, as well as the related molecular regulations 161-165. With the advancement of technologies such as single-cell transcriptomic analysis, the complex heterogeneity and dynamic landscape changes of tumor immune microenvironment (TIME) in NPC has been well described 32-36. For example, lasmacytoid dendritic cells (pDCs), CLEC9A+ DCs, natural killer (NK) cells, and plasma cells were significantly associated with improved survival outcomes in NPC, whereas enrichment of M2-polarized macrophages and LAMP3+ dendritic cells leads to immunotolerance, promoting tumor progression of NPC. The model of “hot” and “cold” NPC generally are classified on the basis of two functional subcategories of TIME cells: hot immune-infiltrated populations such as tumor-infiltrating lymphocytes (TILs), macrophages (M1-polarized), natural killer (NK) cells and mature dendritic cells (DCs), and cold immune-infiltrated cells includes myeloid-derived suppressor cells (MDSCs), mast cells, regulatory T cells (Tregs) and macrophages (M2-polarized). Therefore, predation by the systemic/local immune system is probably an important selective pressure for neoplastic cells in NPC, resulting in immune response. Tumor evolution also enables recruitment of inhibitory immune signals within the tumor stroma, which, in turn, leads to tumor immune escape. For instance, cancer cells are predated by M1 macrophages through phagocytosis; while M2-polarized phenotype (tumor-associated macrophages; TAMs) cooperates with NPC cells each other that encourages the interacted development of both populations 166. In general, the immune system can be converted from a predator into an accomplice (dual role) during cancer progression 167-169. Encouragingly, the exploration of NPC immune environment has advanced targeted immunotherapy of patients turning the TME from immunologically "cold" into "hot". Anti-PD-1/PD-L1 therapy is poised to advance standard for the treatment of recurrent or metastatic NPC (RM-NPC). Several randomized clinical trials in the first-line setting have displayed inspiring improvements in progression-free survival (PFS) with Anti-PD-1/PD-L1 therapy for the treatment of RM-NPC 170-173. In addition, various experimental and clinical studies have explored the use of cytotoxic T cells (CTLs), TILs, NK cells, and DCs in the treatment of NPC patients including refractory and locally advanced stages 174-178. Additionally, researchers have proposed reshaping the systemic tumor immune environment (STIE) to promote immunotherapy efficacy in human solid tumors including NPC 44. With the continuous research and the development of technique, the area of “NPC immunoecology” definitely provides the most promising strategy for the improving survival of patients in advanced stage.

Mutualism, it means that mutually beneficial interactions among individuals of different species 132,151. Sea anemones and hermit crabs is the mutually beneficial sample in nature. Sea anemones are fixed on the shells of hermit crabs to enable them to capture food more effectively; Conversely, sea anemones use toxic prickle cells to protect hermit crabs from natural enemies. The relationship between CAFs and neoplastic cancer cells within a tumor is the good case of mutualism, both of them can get a fitness advantage from the dynamic cooperation 37,179 and even co-evolve during the malignant progression 180-182. It has been well explained in many kinds of cancers, tumor cells strongly influence fibroblast population through the release of several growth factors such as fibroblast growth factor (FGF), platelet-derived growth factor (PDGF), transforming growth factor-beta (TGF-β), receptor tyrosine kinase (RTK) and reactive oxygen species (ROS), all of which contributing to fibroblast activation and proliferation 183,184. In turn, CAFs establish specific communications with tumor cells through secretion of various cytokines, chemokines, exosomes and other effector molecules to mediate the process of ECM remodeling such as increased tissue stiffness in order to promote cooperative mutualism as part of a collective strategy to benefit survival of both populations 185,186. In NPC, extracellular vesicle-packaged EBV-encoded LMP1 can activate normal fibroblasts into CAFs through the nuclear factor-kappa B (NF-kB) p65 pathway, and pre-metastatic niche is formed by activating CAFs in vivo 187. Another interesting report about the relation of mutualism in NPC is that exosomes released from EBV(+) NPC cells could activate YAP1/FAPα axis pathway to promote CAF-mediated characteristics includes fibrosis/remodeling of the ECM and tumor growth, meanwhile induce an immunosuppressive TME that benefits evading predation from immune system 188. On the other hand, the feed-forward loop between tumor cells and “cold ”immune cells is also important mutually beneficial relationship in NPC. One study showed that EBV+NPC cells can recruit monocytes by VEGF and generate a TAM-like phenotype by granulocyte-macrophage colony-stimulating factor (GM-CSF). In turn, TAM can induce EMT and promote NF-κB activation of tumor cells by CCL18 to enhance cancer metastasis 86. The dynamic crosstalk between cancer cells and tumor microenvironment including immune cells in NPC has been well summarized 8,16,189. To my view, such reciprocal interplay of NPC should be better essentially described as population “mutualism” in ecology. Mutualism is the remarkable feature of tumor interspecific relationship between cancer cells and stromal cells (here it means all noncancerous cells within the tumor). Through mutually beneficial exchanges with the extracellular environment and even their coevolution (an important connection between ecology and evolution) 180-182, all of these factors ultimately result in the greatest fitness, that is, maximize tumor survival and reproduction.

Reconstruction of the ECM environment extends to another interesting ecological issue (cancer/NPC stroma ecology). Morphological adaptation is one of the ecological features that populations have an exhibit to adapt to the living environment. For example, in order to accommodate the dry-heat desert environment, cactus plants can show obvious dryophytic characteristics such as the degradation of leaves to thorns. As a matter of fact, through a hybrid discrete-continuum (HDC) model, Anderson AR and the colleagues found that harsh microenvironment conditions (e.g. heterogenous ECM) could exert a dramatic selective force on the tumor, which ultimately grew as an invasive mass with finger-like margins 41. In the leading edge of NPC tissues surrounded by a abundance of stroma, spindle-like cells are prominently found, and often generate fingering edges 14,61,190. We suggest that it should be the more aggressive subtype in NPC, and could be a valuable morphological predictor for NPC concurrence and metastasis. Spindle cancer cells are a good example for the ecological adaptation of tumors. It could be that interactions between normal epithelial cells/tumor cells and the hard ECM environment could shape cancer cell morphological evolution for better-adapted phenotypes 191. A much better understanding of the special ecological mechanisms within different tissue context is required. The cell attractor model was initially constructed to explain the maintenance of normal cell phenotypes 192. The distinct cancer cell “types” (e.g. neoplastic spindle cells) observed in NPC tissues, or the CSCs-like (tumor-initiating) or metastatic states that can be thought as a cancer cell that has maximized its average fitness in NPC, would be such dynamical attractor states. Ernberg I et al. have demonstrated that the rare edge cells might represent cells transgressing the ridge between two basins 193. When the selective pressure of treatment is released after one treatment, the original population can be rebuilt by these temporarily resistant edge cells. Therefore, combination therapy specifically killing rare edge cells that are transiently drug-resistant may be necessary for human cancer including NPC.

**Figure 3:**
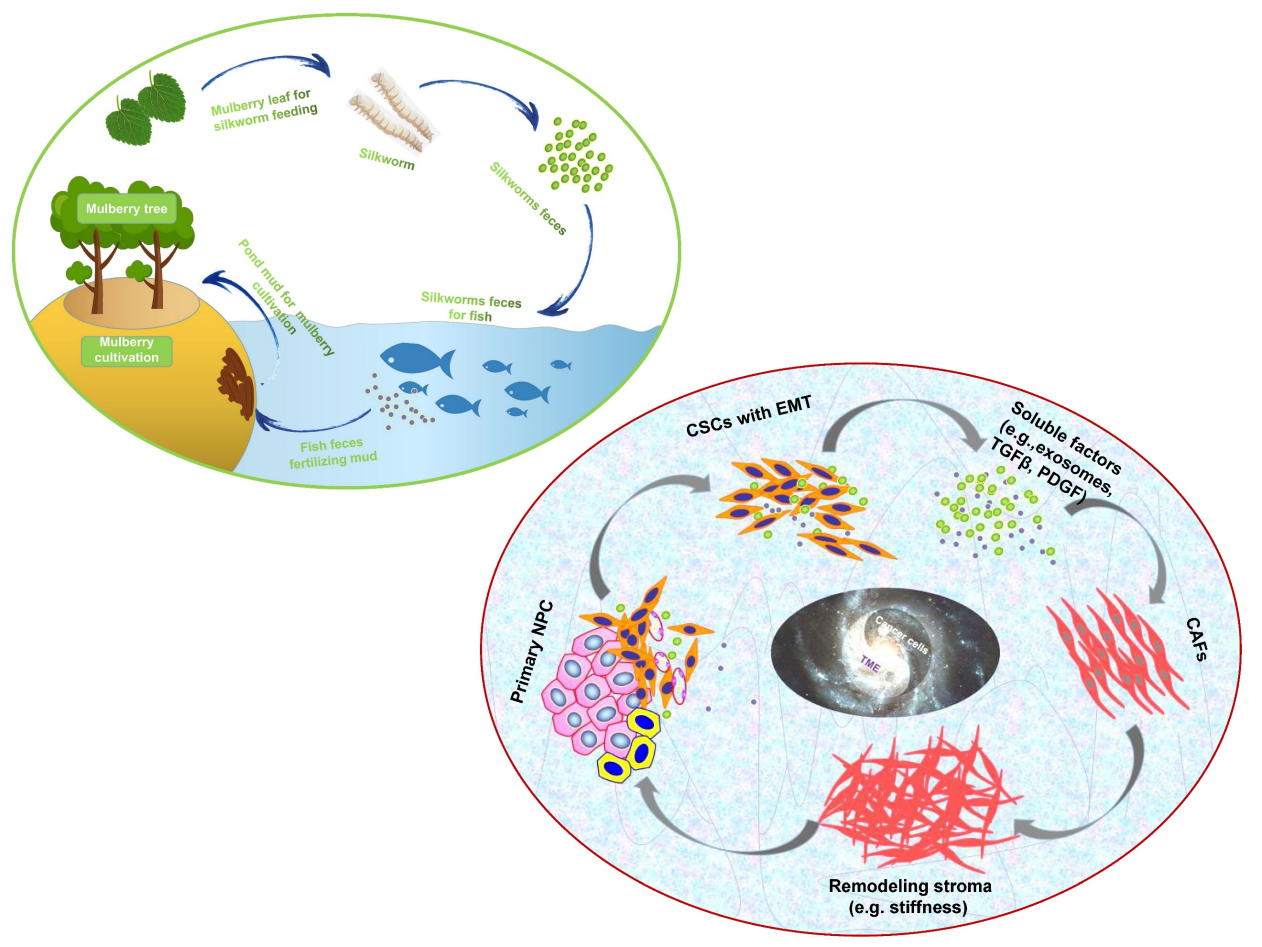
The “mulberry-fish-ponds model” (*top left*) to elucidate the dynamic reciprocity of mutualism between (*bottom right*) cancer cells and TME (e.g. CAFs synthesizing ECM components such as collagen and FN contributes to stromal stiffness, which in turn promotes cancer progression) in cancer ecosystem. Cancer cells and TME work together to build “a community with a shared future” for tumor ecology (ecosystem).

“Dynamic reciprocity” was firstly described in the early 1980's by Bissell MJ and her colleagues to explain the persistent biochemical and biophysical processes that occur between epithelial morphogenesis, breast cancer cells and the ECM 194. The mutualism relationship between these cancer cells and stromal components in NPC should also be recognized as “dynamic reciprocity”. A sustainable agriculture model that is very popular in southern China called “mulberry fish ponds” with a history of thousands of years 195. A special dike-pond mode means that the mulberry plants provide food and habitat for silkworm rearing; silkworms feces and any fallen and rotting mulberry can be used to fertilize fish ponds or as food for the fish; mud from the fish ponds is used for mulberry cultivation. The scientifically based man-made ecosystem - “mulberry-fish-ponds model” (**Figure 3**) could be a typical paradigm to elucidate the dramatic relationship of mutualism between cancer cells and the stromal cells such as immune cells, activated fibroblast, proliferating vascular cells, remodeling ECM and other abiotic factors (e.g. pH, metabolic conditions) in the whole cancer ecosystem. Once tissue-specific gene expression is altered through microenvironmental changes, affected cells may change their behaviors such as cell polarity and junctions, proliferation and invasion, no matter what they carry “cancer mutations” or not. In turn, cancer cells can produce an increasingly abnormal stressful microenvironment through the secretion of the abnormal ECM, and further lead to DNA mutations as a consequence of evolutionary adaptation. The solid evidence about dynamic reciprocity of cancer cell's morphological changes and stromal stiffness can be observed in the pathological specimens of NPC 61. What we can learn from the “mulberry fish ponds” model is that any disrupting in the dynamic interactions of cancer cells and its microenvironment would be an entry point for treatment approaches. Let's take an example. It has been proved that both of mechanical tissue stiffness and metabolic modulation affect directly each other to cause genome-wide changes in epigenetic regulatory states. ECM acts as a metabolic niche in tissue microenvironment, and its targeted therapeutic management might cause metabolic rewiring of tumor cells, and influence crosstalk between nonmalignant and malignant cell phenotypes in the metabolic ecosystem 196. As far as I know, it is the first time to introduce the mulberry-fish-ponds model to the area of cancer ecology.

Parasitism means that one species lives by parasitism in the body or on the surface of another species. Parasitic relationship is based on nutrition and spatial relationship. The adaptations of parasites to parasitic life are various, and at the same time, it will cause host immune response 132,151. The pathogenesis of NPC is closely associated with EBV infection of primary B cells, whereby parasitism is a remarkable characteristic among the interspecific relationships. As time goes by, the evolving cooperation of viral replication with primary B cells ultimately evolves into a mutually beneficial symbiotic relationship, and further promotes host genomic instability and derives somatic mutations and CNVs that are gradually accumulated in nasopharyngeal epithelial cells and often along with the enhancement of adaptative plasticity in response to the TME 6,197,198. Additionally, other pathogens with the parasite relationship including HPV, bacteria or mycetes have also been detected in NPC tissues 199,200. I would like to touch on briefly, the presence of tumor microbiota in NPC tissues was firstly reported in 2021 201, and a retrospective cohort study has recently reported that intratumoral bacterial load is a value prognostic predictor for patients with NPC 202. Though the underlying impacts of bacteria on NPC cells has been carried out 203, more basic investigations and longitudinal clinical studies are expected to be performed in “NPC microecology” as well as other types of human neoplasms 204. We have reasons to believe that, following with the gradual understanding of the symbiosis relation of microbiota and NPC, as well as the mechanism of microorganisms in the occurrence and prognosis of this disease, and with the emerging development of small molecule microbiome modulator (SMMM), the research of NPC microecology will provide effective strategies for the prevention, diagnosis and treatment of NPC patients.

Population competition includes intraspecific competition and interspecific competition; the former refers to competition among individuals of the same species, and the latter means that competition among two or more species of organisms that are adversely affected by the use of common resources 46. For example, barnacles competing for space on rocks belongs to intraspecific competition, whereas the competition between Salvelinus malma and S. leucomaenis, Asterionella formosa and Synedra ulna fall inside interspecific competition 132. Cancer cell competition between tumor cells themself, or tumor cells and their microenvironment withing a tumor mass has been widely explored. Aggressive cancer cell populations achieve high fitness status and competitive abilities by displaying traits such as increased proliferation, de-differentiation and stemness governed by cell-competition genes P53, Myc and several signaling networks including the Warts/Hippo, PI3K/Akt/mTOR, JAK/STAT, and Wnt pathway 205,206. Spindle cells in NPC tissues having the EMT and stem cell-like features could be the more competitive cancer cell populations 14,61. In some cases of NPC biopsy, areas of coagulative necrosis are observed and can be extensive 12, which might be partly due to increased competition of cancer cells within a rapidly growing tumor encountering with the harsh conditions such as limited space, hypoxia and insufficient nutrient resources. Up to now, however, the competitive interactions and cellular/molecular competition pathways between tumor cell subpopulations or with the stromal tissues remain largely unexplored in NPC.

Heraclitus once said, "you cannot step into the same river twice”. From the above, we believe that the relationship between cancer cells and tumor microenvironment is not static, ecosystems themselves do not function in isolation. They grow and decline, transform, cooperate, select and co-evolve with each other in the whole pathological ecosystem spatially and temporally, just like the principle of “Yin-Yang theory”, which is created by the ancient Chinese people used by practitioner of Chinese medicine to explain the organizational structure, physiological function and pathological changes of the human body, and to guide the diagnosis and treatment of diseases.

## Tumor-host interface as the ecological transition zone

**Figure 4:**
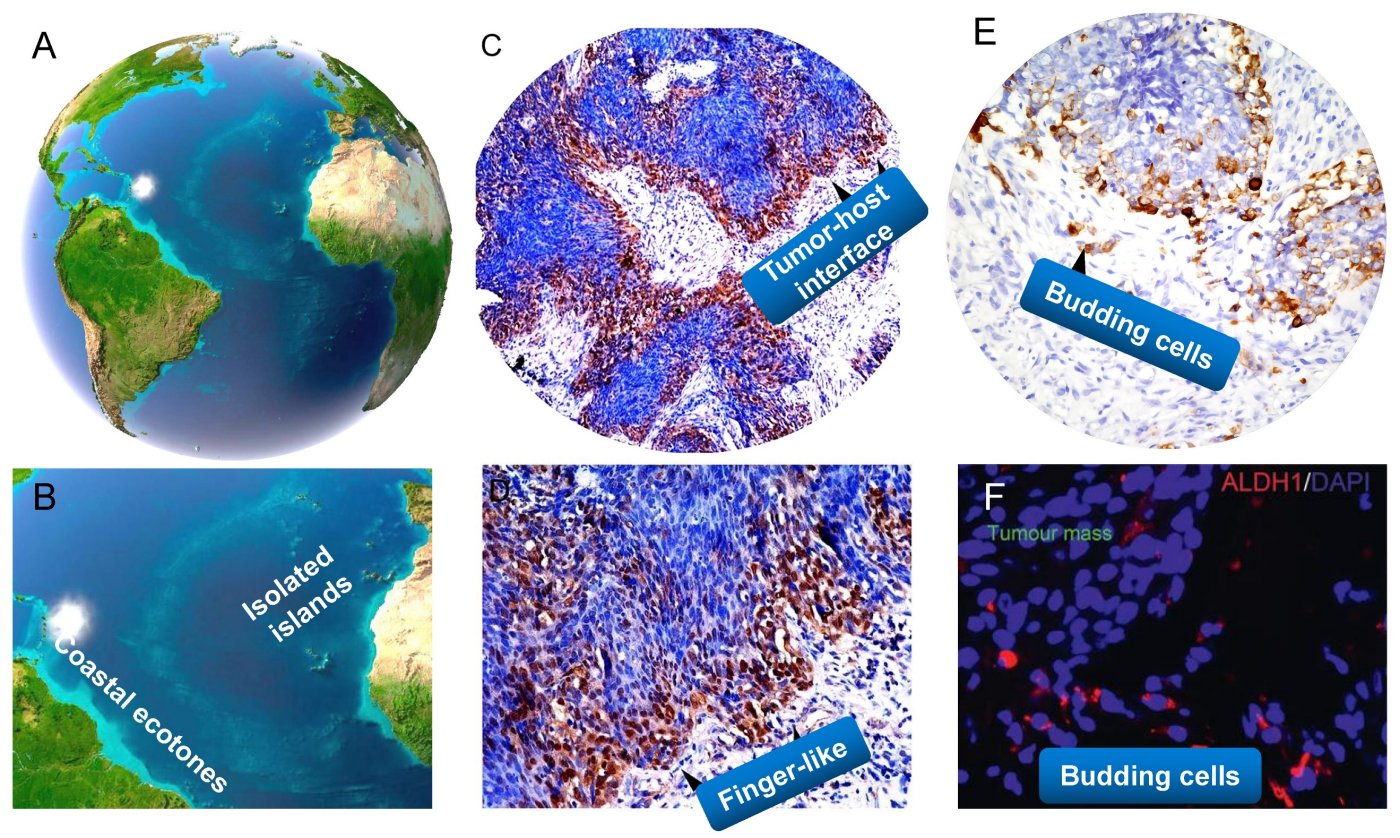
A close analogy of tumor-host interface and budding cells in NPC with the ecological nature. Land/sea transitional zone and isolated islands on earth **(A,B)**. Tumor-host interface as the ecological transition zone, it often takes curved and finger-like shapes **(C,D)**. Tumor buddings are similar to little islands separated from the continent **(E, F)**. (**A,B** adapted from image.baidu; **C-F** adapted from our previous studies, Luo WR et al, (Ref.136,229) ).

Ecological transition zone (ecotone) is the transition zone along the edges of two adjacent ecological communities, the place where one ecological community meets another 132,151. Ecotone is a special area with the characteristics of adjacent communities and its own characteristics 207. It has several main characteristics, for example, a joint action and conversion area of various elements, with strong interaction and a greater biodiversity of species than either of the two separate ecosystems; the ecological environment is weak to resist interference and external forces; the ecological environment changes fast and has strong spatial dispersal ability and greater productivity. Accumulating studies have shown that tumor-stroma interface withing a tumor is the crucial part of the tumor 126. Its interface with the stromal components, also termed as the invasive tumor front (ITF), which is commonly defined as the three to six layers of caner cells at the invasive margin or the disseminated tumor groups between tumor and host tissue. The interaction of cancer interface with the surrounding stromal components attracts much attention in modern cancer biology 137, 208. In my standpoint, tumor-host interface could be considered as “ecological transition zone” where tumor cells meet so closely with the stromal components (**Figure 4**). This idea remains largely unexplored in the past decades.

Tumor-host interface shares the similar features with the ecological transition zone. Such is the case. Tumor-host interface has been proved to contain the most useful prognostic information for patients due to the acquisition of migratory and invasive features, secretion of proteolytic enzymes, reorganization of the extracellular matrix 209,210. Moreover, newly generated blood vessels, TAMs, CAFs, and other stromal cells are highly accumulated at the tumor-host interface 211,212. More importantly, such interface is a area inhabited by a particular species of highly resistant CSCs and the niches of TME 211. However, up to now, we don't know much about tumor-host interface in NPC. This concepts as well as "CSCs niche" and "migratory CSCs" were formally introduced in the research area of NPC 136,190. We found that stem cells-like features such as high expression of OCT4 and Nanog were more frequently located at the tumor-host interface, and correlated significantly with aggressive behaviors such as lymph node metastasis and distant metastasis of NPC patients 136. In addition, we indicates that cancer cells in the invasive front of NPC acquiring stem cell-like features should be maintained by vascular niches. Mathematics of tumor growth also shows that a tumor shaped like a solid sphere in which the stem-like cells are arranged at its surface only 126. Therefore, these findings imply that tumor cells localized within this region are more prone to promote adaptive responses to changes of the environment, thus evolve more capacity to promote drug resistance and tumor relapse. It has been found that tumor cells at tumor-host interface have transformed from an epithelial (low mobile/invasive potential) to a mesenchymal (high mobile/invasive potential) phenotype (EMT) 213. A single-cell transcriptomic analysis of primary and metastatic tumor ecosystems in head neck cancer showed that cells expressing the partial EMT (p-EMT) program spatially localized to the interface of primary tumors, and predict nodal metastasis 214. These p-EMT cells do not represent cloning regionalization of mutationally altered cells but represent epigenetically regulated plasticity of the fittest cells selected for invasive and malignant progression 42. As we have described, cancer cells are often present with spindle-like morphology at tumor-host interface of NPC. Moreover, EMT and hybrid E/M states such as membranous/cytoplasmic E-cadherin and nuclear vimentin and Snail, and CSCs marker ALDH1 are aberrantly expressed in the leading edge, all of these proteins are valuable predictors of poor survival in NPC 14,53,215-217. Similarly, one impressive report recently has also found that sarcomatoid carcinoma (SC) subtype (that is we call neoplastic spindle cells here) of NPC exhibits enriched EMT and invasion promoting genes, and significantly correlates with their morphological features 218. As may be inferred from this, these malignant cells in tumor-host interface exhibiting mesenchymal-like properties should be more likely to detach from the primary sites and diaspora at the early stage. Emerging evidence has showed that cancer cells with EMT properties induced by EBV-related factors such as LMP1, LMP2A and EBV-miR-BART7-3p can directly contribute to the metastatic progression of NPC *in vitro* and *in vivo* 54,65,219,220. In summary, tumor-host interface of NPC (cancer) representative of migratory/metastatic CSCs is the ideal battleground corresponds to a tumor microenvironment, and phenotypic plasticity change is one of the remarkable features of tumor-host interface area.

As described above, at tumor-host interface of tumor ecosystem, you can find that tumor‐promoting immune cells or vascular cells are particularly concentrated, and CSCs-like cancer cell populations with invasive features are specially adapted to the conditions of the transition zone. This interesting phenomenon is referred to the “edge effect” 151, which is an ecological concept that describes how there is a greater diversity and increased number of population species in the transition zone. The enhanced diversity and productivity deriving from the edge effect are commonly observed in the Nature 221,222. Wetland ecology and mangrove ecology (land/sea transitional zone) are typical representatives of the most highly productive natural systems. All of the resources such as water, humidity, air light and temperature, as well as the massive movement of nutrients and energy exchange and interact with each other in their interfaces. Indeed, tumor-host edge is such a battleground of fierce fighting in which there is repeated crosstalk between tumor cells and the stroma (e.g. stromal cells together with ECM and released factors from cancer cells such as exosomes, cytokines and growth factors), thus results in evolutionary plasticity of both cancer cells (e.g. changes in plasticity of cell junctions and EMT, transition from collective cell migration to individual cell migration) and the host environment (e.g. degradation, deposition and remodeling of ECM, alteration of ECM stiffness and porosity), and finally leads to invasive and metastatic progression of the tumor. It is therefore not difficult to see that a greater number of mutualism behaviors among the elements are present at the edges of cancer.

Nature does not like to be uniform. The meandering river, zigzag coastline, curved rivers and uneven surface are all its masterpieces. These diverse shape patterns of edges generate greater diversity of species and increase productivity 132,151. For example, the zigzag coastline of mainland increases the precipitation of the land, and diversity of nutrients and organisms; the meandering river flows through a wider area to irrigate more plants; the road of life is also as winding as a river. Similarly, the macrocosm pattern are reflected in structure of the human body (e.g. our blood circulation, intestines, or even inner mitochondrial membrane), for instance, curved blood capillary that blood can directly exchange nutrients and metabolites with tissues more effectively than if they are running in a straight line. As expected, when viewing at microscopic resolution, we can also see that the patterns of tumor-host interface in cancer tissues including NPC are not aligned, and they usually take winding, finger-like or other irregular shapes 14,136,215. I suppose that the adaptive potentials, such as sustaining proliferative signaling of tumor cells in the interface that serves as an ecological factor to the surroundings, are maximized by the curved patterns of shape, just like the finger-like "Pillars of Creation" in which a lot of newborn stars bred at its front. But then, another question is coming. Can we take the irregular phenotype of cancer cells back to normal? The answer is yes. The phenotypic reversion of cancer cells in 3D context actually has been well investigated by Bissell MJ's group 223,224. They found growth arrest and polarity restoration in disorganized breast cancer T4-2 cells in response to inhibition of β1-integrin. Another interesting study by Ewald AJ et al. in 2013 showed that, K14^+^ cells were enriched at the tumor-host interface leading collective invasion and lung metastasis in primary human breast tumors in 3D culture and *in vivo*. In contrast, knockdown of either K14 or p63 was sufficient to block the invasive protrusions of cancer cells subsequently with rounded cell borders 225. Deep investigating the molecular mechanisms of tumor-host interface is crucial for the development of “anti-ITF” ecological strategies in human cancers.

## Tumor buddings as ecological islands separated from the continent

Tumor budding (TB) is an emerging prognostic biomarker in colorectal cancer (CRC) and other solid cancers 224-226. In particular, tumor budding has been listed as an additional prognostic factor in the 2017 UICC/AJCC TNM classification and is included in the 2019 WHO classification of tumors 227, 228. Our previous study firstly showed the presence of tumor buddings, and these cells migrated collectively or individually and correlated with aggressive tumor behaviour in NPC 229. Tumor buddings should be considered as ecological separated islands, and both of them shared the similar features. They have similar geographical locations, tumor buddings are generally defined as isolated single cancer cells or clusters of up to four cancer cells detaching from the tumor-stroma interface, and is thought to be the first step of tumor dissociation from the main tumor mass 226, whereas an isolated island is a small land separated from the mainland surrounded by water. More importantly, they all have important and unique features. The biological characteristics of isolation has a profound impact on the population and community living on the island 132. Physical distance and connectivity between the mainland and isolated areas might contribute to population dispersal and extinction rates. Just like isolated islands of low connectivity with the mainland, tumor buddings have the spatiotemporal isolation of areas and their specialized diversity. A recent review has postulated that the whole tumors are evolutionary island-like ecosystems that drive cancer cells' migration 230. It is noteworthy that tumor budding has been proved to be strongly associated with EMT property in a variety of cancers such as colon cancer, lung caner, pancreatic cancer and oral cancer 231-234. Photomicrograph of histologic specimens in NPC show that spindle-like morphology is frequently exhibited in tumor buds, and these cells possessed the EMT and CSCs characteristics in cancer tissues 61,229, which indicating that budding cells can acquire more invasive and unlimited proliferated abilities to approach the vascular architectures for intravasation and to seed the new populations. We demonstrate that tumor buddings could be valuable morphological predictors for NPC early invasion.

Therefore, the interaction between tumor buddings as a tumor-related factor and vascular cell, fibroblasts and immune cells as a host-related factor forms islands-like systems. Tumor buds can be seen as the heavy cavalry of cancer, and its ideal battleground is tumor-host interface (**Figure 4**). However, I have to say that, it is hard to distinguish budding cells from fibroblasts or other mesenchymal cells by haematoxylin and eosin (HE) staining or even pan-CK staining in NPC tissues, because these budding cells migrating the stroma commonly have a similar fibroblast-like phenotype, and the context is always complex with a dense infiltrate of lymphocytes and plasma cells. 3D cell culture and organiods might be ideal tools for better elucidating morphogenesis and ecological interrelations of tumor buds in NPC. A better understanding of the molecular and pathogenetic mechanisms disclosing tumor budding might contribute to the development of “anti-budding therapies” 226.

## Selection driving factors and ecological therapy in NPC

Darwin's natural selection is an ecological process leading to adaptive evolution, which is occurred by various biotic and abiotic factors together in the complex habitats. The organisms constantly absorb substances from the habitat environment to meet their needs for survival and development, but is also changing the environment. In turn, the environment changes feed back into the organisms, and make the latter adapt to the new environmental changes 143. Any environmental factor (evolutionary driver) is the cause of natural selection, and differential survival and reproduction is the result for natural selection. The integration of ecological and evolutionary aspects holds promise for understanding evolution and metastatic progression of NPC, and the primary classification of selection factors is briefly discussed here.

The recognition of the importance of tumor microenvironment, ECM modification, niche construction and ecological relationships among cancer cells, stromal biotic/abiotic components has resulted in a promising cancer treatment so-called “ecological therapy”, which was initially proposed to for the therapeutic development of prostate cancer metastases 235. If we consider the context of darwinian evolution, the most efficient way to kill a species is to destroy its niche by altering the surrounding environment. The ecological “habitat” is an area inhabited by a particular species of animal, plant, or other type of organism 236,237. For example, an analogy to riparian ecosystems is drawn in human cancers, and tumor vascular network is thought to be a main cause of intratumoral heterogeneity 236. Habitat destruction such as hyperthermia, antiangiogenic therapy, pH alteration, matrix metalloproteinase inhibitors could lead to causes of species extinction, and it might be analogous cancer treatments 135. For example, we can unitize the principle of ecologic traps to induce cancer cells to a designate habitat where they can be better exposure for antigen presentation of cells of the immune system 76. Here I want to talk a little bit about the selection driving factor “heat”, which has been recognized to be a crucial microenvironmental and epigenetic factor as to biological development 238,239. A combination of cytotoxic drugs and hyperthermia have additive effects through the increase in ROS and subsequent DNA damage and apoptosis. Hyperthermia is especially effective in hypoxic and nutrient deprived areas of the tumor, and leads to an increased expression of immune checkpoint molecules (ICMs) when combined with radiotherapy. A model indicates that hyperthermia during the first radiotherapy fractions seems to be more effective in an artificial immune-tumor ecosystem 240. In the last few decades, hyperthermia (41-43°C) has been a successful treatment adjunct strategy in many types of cancer such as ovarian cancer, colorectal cancer, bladder cancer and breast cancer 241-245. A new study also showed that whole-body hyperthermia (WBH) combined with chemotherapy and IMR treatment can improve the 5-year overall survival (OS) of patients with advanced NPC 246. Recently, our results have revealed that hyperthermia alone or combination treatment of oridonin dramatically can increase the killing effect on NPC cells and CSCs-like population 247. Moreover, hyperthermia remarkably improves the sensitivity of NPC radiation-resistant cells and CSC-like cells to radiotherapy.

The early attempt of an ecologically-inspired approach known as “adaptive therapy” looks indeed very promising, in which cancer is treated by alternating different drugs that can suppress growth of resistant populations and avoid/delay drug resistance 45. Beyond that, other ecological approaches include adaptive immunotherapy targeting predation, oncolytic virus therapy as to parasitism and continuous pressure by metronomic therapy also provide promising potentials for the treatment of NPC patients.

## Conclusions and perspectives

Gorky once said that there is no limit to the bold activities of science, nor should there be any limit. Over the past decades, a series of theories and results as to cancer ecology presented by a number of theory-minded cancer scientists have been put forward to explain carcinogenesis. As long ago as the early of 1960s, Smithers DW regarded cancer as a disease of organisation, in which “an abnormal cell may produce a clone of cells reacting abnormally with their environment, and environmental stress may disorganise the behaviour of cells within a sphere” 7. In the 1970s, Nowell PC published the pioneer work “the clonal evolution of tumor cell populations” 27. In the 1980s, the concept of a tumor as “an ecosystem” was proposed by Heppner GH 248,249. In the 1990s, Pienta K J et al. suggested that cancer can be viewed as “a complex adaptive system” 250, and firstly proposed the strategy “ecological therapy” for cancer in 2008 235. Maley CC et al. demonstrated cancer as “an evolutionary and ecological process” in 2006 37, and they classified the evolutionary and ecological features of neoplasms by Evo- and Eco-indices in 2017 140. In 2018, Greaves M pointed out that “nothing in cancer makes sense except in the light of evolution” 18. In 2021, Massagué J et al. discussed the nature of metastasis-initiating cells (MIC) and their ecosystems for innovative treatments of metastatic cancers 251. At present, I declare that human cancer includes NPC is a multidimensional spatiotemporal “ecological and evolutionary unity” pathological ecosystem, “cancer is not a genetic disease but a disease with ecological and evolutionary unity”.

Treating cancer as “unity of ecology and evolution”, and application of evolutionary and ecological principles to cancer prevention and treatment should be deeply rooted in our mind. A comprehensive understanding of tumor ecology warrants further study although it has been gained increasing attention. In particular, nine overarching eco-evolutionary questions have been recently putted forward by Dujon AM et al. 252. Additionally, we should be aware that current study of “cancer ecology” is mainly limited to “general tumoriecology”, and the area attempts to apply the general principles and diverse models of ecology to the research of cancer ecology. Although they share the common “hallmarks of cancer”, tumors deriving from different systems and different sites should have their specific ecological and evolutionary properties. For example, distinct cancer sites such as pancreatic cancer, lung adenocarcinoma, glioblastoma and skin melanoma have different levels of tissue architectural heterogeneity, stroma to tumor ratios and fibrotic reaction 169; colorectal cancer and gastric cancer belong to the gastrointestinal neoplasms but reside in different microbiota niches; even incellular subsets of hematologic neoplasms such as chronic lymphocytic leukemia (CLL) and myelodysplastic syndromes (MDA) compete for marrow niche occupancy. Therefore, it is time for us to go deep into the systematic research of “system/organ tumoriecology”.

Ecological pathology: The "father of modern medicine” William Osler once said, “as is our pathology, so is our practice”. With the development of new theories and techniques, the research of various branches of pathology is so all-encompassing at present. What I want to emphasize here is that, we need a dynamic ecological view to truly understand and integrate human pathology, and it is essential to apply the ecological (-evolutionary) principles and approaches to study the etiology, pathogenesis, pathological changes and outcomes of human diseases. I collectively call this research subject as “ecological pathology”. Some inspiring explorations have been made in the branch of this field 253-256. For example, we should apply quantitative, spatially explicit methods from landscape ecology to define the heterogenous biological processes of cancer cells in clinical pathological samples 256. On the other side, we should study ecology of diseases from all of aspects of pathology such as histopathology, cytopathology, molecular pathology, ultrastructural pathology or computational and artificial intelligence (AI) pathology. According to the area of ecological oncology, the histopathology and related pathological techniques such as tissue clearing and 3D construction are important supports for its systematization and visualization 257. It is interesting to start from a pathological perspective to think about tumor ecology. For example, the ecological dynamics of tumor-host interface or budding cells across space and time, or a special pathological phenomenon or (sub)structure such as cell-in-cell/tunneling nanotubes/liquid-liquid phase separation (LLPS) 258-260, all of those are worthy of further ecological investigation. Not only that, this area should be jointed with emerging technologies in other medical subjects. Interestingly, computed tomographic (CT) imaging can identify intratumoral Darwinian dynamics before and during the treatment of cancer patients 261. Additionally, MRI can rapidly estimate intratumoral evolution of the molecular properties of cancer cells, which might be the equivalent of satellite images in landscape ecology 262. Such quantitative analysis of radiologic images for explicit measure of temporal and spatial evolutionary dynamics of cancer cells within their image-defied habitats may be termed as “ecological radiology”. Hence, it is hard to image what kind of sparks will the combination of ecological perspective of radiology-pathology bring about.

Multidimensional tumoriecology: Single-cell genomic, transcriptomic and epigenomic sequencing methods have grown rapidly over the past decade. These works are mainly depicted an interactive network among cancer cells, stromal cells, and immune cells in tumors. However, the complexity of ecosystem is far more than that, as described above. More importantly, disassociated tumor tissues have lost information about the crucial spatial location of cells in primary tissues by single-cell seq technology. We should remember that cancer is an eco-evolutionary process driven by the spatial feedback between evolving tumor cells and microenvironmentally temporal selection. In recent years, oncologists and biologists tried to uncover the ecosystem in several types of human cancer through single-molecule fluorescent in situ hybridization (smFISH), the RNAscope assay, and even by spatial transcriptomics 253. This “spatiotemporal tumoriecology” may lead to a comprehensive decoding of cancer and TME ecosystem in situ tissue samples. However, one of the technical challenges is that these current spatial omics-based images are still remained in the 2D histological stage that fails to possess the spatial organization and microenvironment of cancer tissues. It is hard for us to imagine the significance of studying landscape ecology without considering the interaction between spatial heterogeneity and ecological processes. Tumor is like a solid sphere rather than a planar 145. Just as Bissell MJ declared in 2017, we should “goodbye flat biology"-time for the 3rd and the 4th dimensions” 263. This is a critical point in the history of cancer research-we are at a crossroads, that is, an important juncture in combination of the perspective of spatial distribution, temporal variation of the populations, as well as a multi-dimensional perspective to comprehensively exploring of tumor ecology or understanding of the true colors of cancer (multidimensional spatiotemporal oncology/tumoriecology). With the integration of emerging approaches 264-269 such as the 3rd or 4th dimensions of the spatial omics and pathological tissue and cellular reconstruction, which could be a powerful platform for our understanding of the evolutionary and ecological process of human cancers. I would like to point out in particular that multidimension is a broad term here, it also includes cross-level of organization (gene, genome/epigenome/proteome/metabolome, organelle, cell, tissue, organ, system, organism, biosphere, etc.). Tumor ecosystem has the characteristics of hierarchical system in space. Cancer research has also been proposed to be viewed through the theory dimension, the systems dimension, the time dimension and the micro-/environment dimension 270. (Again, I think this holds for the study of cancer ecology).

Integrated tumoriecology: Tumor ecology is indeed a new interdisciplinary field, multidisciplinary fields such as physics, chemistry, computational modeling, biomechanics and ecology needs to be integrated 252. It has been proved that microbiomes/immune cells are important to the pathogenesis and prognostic outcome of patients, whereas the ecological and evolutionary roles of the microbiomes/immune cells, and how their eco-evolutionary dynamics determine host fitness in various cancers are still poorly understood. Therefore, investigators are encouraged to organize and develop the appropriate multidisciplinary team, for example, pathologists, modelers, ecologists, bioinformaticians, oncologists, geneticists, evolutionary ecologists, physicists, mathematicians, microbiologists and virologists, with the goal of integrating knowledge across disciplines to drive novel and comprehensive insights into the theoretical and experimental development of tumor ecology. A broad cross-disciplinary collaborations network in tumor ecology is formed between people engaged in scientific research because of their communications and collaborations in scientific researches, academic researches and practice, which is termed here as “integrated tumoriecology”. Ultimately, the integration of oncol-ecology will have a bright future in the greater benefit of patients.

**Figure 5:**
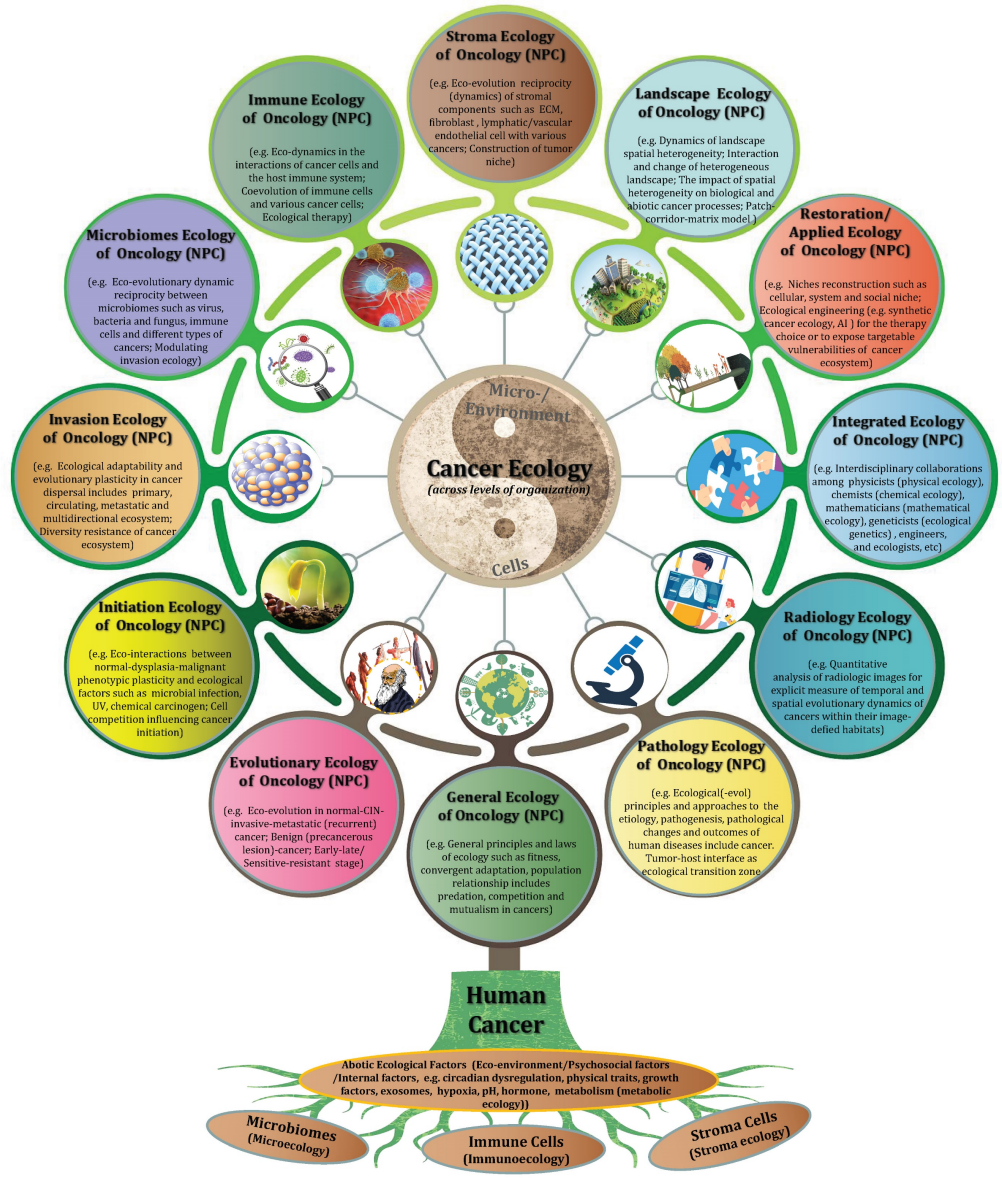
Human cancer ecology tree. The cancer ecology tree is unprecedentedly constructed to elucidate the framework and research direction of cancer ecology. The nature of human cancer includes NPC is an “ecological and evolutionary unity” disease.

In the end of this thesis, I advocate that the insight into the nature of NPC and according conceptual ideas can be generalized to all (cancer) cell types in our body. The role of DNA is not for reprogramming cells. The description such as “Cancer is a genetic disease” or “Cancer is caused by mutations in oncogenes” has overlooked an important fact that any cell behaviors is caused by numerous coordinated regulatory events that are heavily reliant on functional integration not only within single cells but also within tissues and the organism as a whole 270. The initiation and progression of human cancer (e.g. the status from the normal to diseased, early to late, local to diffuse), for example that we are familiar with, chronic cervicitis-cervical intraepithelial neoplasia (CIN)-micro-/invasive cervical cancer-metastasic cancer, acute/chronic viral hepatitis-early/late cirrhosis-primary/metastatic liver cancer and normal-polyp(adenoma)-different stages of colon cancer, which can be considered as an ecological disease, that is a multidimensional spatiotemporal ecological/-evolutionary process as a whole. In such an eco-pathological system with disorderedcellular tissue architecture, parenchymal cells dynamically interplay with their specific micro-/environments such as microorganisms, immune cells, stromal cells and abiotic factors (along with the communication of energy, message and matter), adapt to each other and even co-evolve in a cross-level manner. Emerging evidence reveals that microbiomes have an ecol-evolutionary role of modulating the functions of host innate immune responses in the development of human diseases including cancer 271,272. We can estimate optimistically, if regarding cancer as a pathological ecosystem, some newly arisen subjects and technologies, for example, applying the knowledge system and research methods of synthetic biology may be of great help with the damaged ecosystem such as the reconstruction/healing of diseased cellular niches, and further provide more promising treatment strategies for patients in the future (I'll refer to this as synthetic cancer ecology/synthetic ecological therapy). As indicated below, the cancer ecology tree is comprehensively constructed to show the complete frame and research interests of cancer ecology (**Figure 5**). The somatic mutation paradigm is still the mainstream in current cancer research, however, just like the famous cancer biologist Bissell MJ et al. said, the time has come for us to “rethink cancer” 270.

## Abbreviations

NPC: nasopharyngeal carcinoma
EBV: epstein-Barr virus
DNKC: differentiated nonkeratinizing carcinoma
UDC: undifferentiated nonkeratinizing carcinoma
CSCs: cancer stem cells
LRC: label-retaining cell
SP: side population
ALDH1: aldehyde dehydrogenase 1
VEGF: vascular endothelial growth factor
EV: extracellular vesicles
ECM: extracellular matrix
EMT: epithelial-mesenchymal transition
p-EMT: partial EMT
EnMT: endothelial-mesenchymal transition
MRI: magnetic resonance imaging
DTCs: disseminated tumor cells
MRD: minimal residual disease
TACE: transcatheter hepatic artery chemoembolization
TIME: tumor immune microenvironment
pDCs: lasmacytoid dendritic cells
NK: natural killer
TILs: tumor-infiltrating lymphocytes
MDSCs: myeloid-derived suppressor cells
Tregs: regulatory T cells
TAMs: tumor-associated macrophages
CAFs: cancer-associated fibroblasts
CTLs: cytotoxic T cells
RM-NPC: recurrent or metastatic NPC
FGF: fibroblast growth factor
PDGF: platelet-derived growth factor
TGF-β: transforming growth factor-beta
RTK: receptor tyrosine kinase
ROS: reactive oxygen species
GM-CSF: granulocyte-macrophage colony-stimulating factor
NF-kB: nuclear factor-kappa B
HDC: hybrid discrete-continuum
ITF: invasive tumor front
TB: tumor buddings
CRC: colorectal cancer
HE: haematoxylin and eosin
WBH: whole-body hyperthermia
CLL: chronic lymphocytic leukemia
MDA: myelodysplastic syndromes
CT: computed tomographic
SmFISH: single-molecule fluorescent in situ hybridization
MIC: metastasis-initiating cells
SMMM: small molecule microbiome modulator
